# Treatment-free remission after third-line therapy with asciminib in chronic myeloid leukemia with an atypical e19a2 *BCR::ABL1* transcript and T315I mutation

**DOI:** 10.1038/s41375-024-02327-2

**Published:** 2024-07-04

**Authors:** Philipp Ernst, Jenny Rinke, Georg-Nikolaus Franke, Frank Dicker, Torsten Haferlach, Thomas Ernst, Andreas Hochhaus

**Affiliations:** 1 Klinik für Innere Medizin II, Universitätsklinikum Jena, Comprehensive Cancer Center Central Germany, Campus Jena, Jena, Germany; 2 Klinik und Poliklinik für Hämatologie, Zelltherapie, Hämostaseologie und Infektiologie, Universitätsklinikum Leipzig, Comprehensive Cancer Center Central Germany, Campus Leipzig, Leipzig, Germany; 3 MLL Münchner Leukämielabor, München, Germany

**Keywords:** Chronic myeloid leukaemia, Epidemiology

Chronic myeloid leukemia (CML) is characterized by a reciprocal translocation between chromosome 9 and 22 in the hematopoietic stem cell that results in formation of the Philadelphia chromosome (Ph), encoding the *BCR::ABL1* fusion gene [1]. The formation of the corresponding   BCR::ABL1 oncoprotein causes the depletion of the N-terminal cap of Abelson murine leukemia viral oncogene homolog 1 (ABL1), which under physiological conditions binds in the myristoyl pocket of the C-terminal lobe of the kinase domain and thereby negatively regulates its activity [2]. Loss of ABL1 autoregulation contributes to the constitutive activation of BCR::ABL1, driven by homo-oligomerisation of BCR::ABL1 mediated by the coiled-coil domain of the breakpoint cluster region (BCR) protein [3], which in turn induces uncontrolled proliferation and survival of leukemia stem cells. Apart from the typical *BCR::ABL1* transcripts e13a2 and e14a2, less than 2% of patients express atypical transcripts such as e1a2, e8a2, or e19a2. In these cases, however, reliable monitoring by routine real-time quantitative polymerase chain reaction (RT-qPCR) is not feasible, therefore an assessment of the individual molecular response with specific RT-qPCR primers is recommended [4, 5].

The advent of tyrosine kinase inhibitors (TKIs), which competitively disrupt enzyme activity through binding on the adenosine triphosphate (ATP)-binding site of BCR::ABL1, has substantially improved outcome of CML patients and is now standard of care. For first-line treatment of CML in chronic phase (CML-CP), imatinib as well as second-generation TKIs (2GTKIs) dasatinib, nilotinib, and bosutinib are recommended [6]. Compared to imatinib, 2GTKIs achieve earlier and deeper molecular response, permitting treatment-free remission (TFR) more frequently. However, off-target inhibition may be associated with a distinct side-effect profile [6]. In the European Stop Tyrosine Kinase Inhibitor (EURO-SKI) trial, 46% of patients showed a major molecular response (MMR) 3 years after discontinuation of treatment. So far, due to the need for standardized assessment of residual disease, only patients with typical transcripts have been investigated in discontinuation studies. The e14a2 *BCR::ABL1* transcript is favorably associated with the success of long-term TFR [7].

Resistance and intolerance to ATP-competitive TKIs have been described in about 25% of cases [8, 9]. T315I mutations occur in around 5% of patients treated with 2GTKIs and confer resistance by restoring or increasing ABL1 kinase activity [9, 10]. Ponatinib, a third-generation TKI, and allogeneic stem cell transplantation were for many years the only successful treatment options [6, 11]. Recently, with asciminib, a TKI with a new mode of action has become available, specifically targeting the ABL myristoyl pocket (STAMP) independent of ATP site mutations [12]. Due to its superior efficacy and enhanced tolerability, asciminib was recently approved for the treatment of chronic phase CML after failure or intolerance of at least two TKIs [12], or, in some countries, for patients with T315I mutation [13].

Here we report on a young patient with CML who was, after TKI resistance with T315I mutation and subsequent intolerance to ponatinib, reluctant to attempt allogeneic stem cell transplantation. The patient decided instead for participation in a phase I trial with asciminib and achieved a sustained treatment response with undetectable e19a2 *BCR::ABL1* transcripts, permitting a successful discontinuation attempt. The clinical trial has been approved by the responsible ethics committee. Patient samples investigated in this study were obtained after informed consent and according to the Helsinki Declaration. The patient supported the analysis and agreed with the publication.

**Fig. 1:**
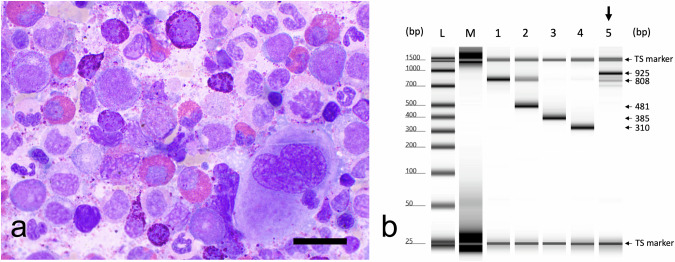
Bone marrow aspirate (May Gruenwald–Giemsa stain) and qualitative multiplex PCR for *BCR::ABL1* transcripts [14] at diagnosis in November 2011. (**a**) Bone marrow shows hypercellularity with maturing myelopoiesis and increased basophil and eosinophil granulocytes consistent with CML in chronic phase, scale bar = 10 µm. (**b**) Qualitative multiplex PCR revealed in lane 5 the atypical transcript e19a2 of the patient (925 bp). *BCR* bands (808 bp) are internal positive controls in lanes 1 to 5. L- ladder (HSD1000 tapestation). M—DNA free master mix; lane 1—negative control; lane 2—SD1 cell line (*BCR::ABL1* transcript e1a2, 481 bp); lane 3—K562 cell line (*BCR::ABL1* transcript e14a2, 385 bp); lane 4—BV173 cell line (*BCR::ABL1* transcript e13a2, 310 bp); marker—upper and lower tapestation marker.

**Fig. 2:**
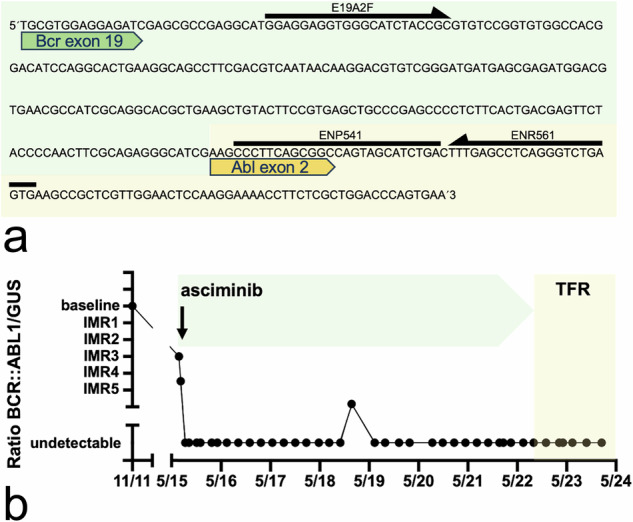
Base sequence of *BCR::ABL1* e19a2 transcript of the patient (exemplary excerpt) and course of individual molecular response (IMR). (**a**) Following Sanger sequencing of the e19a2 transcript, the forward primer E19A2F (5′-GGA GGA GGT GGG CAT CTA CCG-3′) and the conventional reverse primer ENR561 (5′-CAC TCA GAC CCT GAG GCT CAA-3′) with the ENP541 (5′-CCC TTC AGC GGC CAG TAG CAT CTGA-3′) probe were used for MRD analysis by RT-qPCR. (**b**) To calculate the individual molecular response (IMR), log reduction during treatment with asciminib was determined by comparison to the ratio *BCR::ABL1/GUSB* [4]. ENP European network TaqMan probe, ENR European network reverse primer, Bcr breakpoint cluster region, Abl Abelson murine leukemia viral oncogene homolog; GUSB - beta-glucuronidase; TFR treatment-free remission.

In November 2011, the 33-year-old male patient was diagnosed with CML with leukocytosis of 99 × 10^9^/L with 21% basophils, 13% blasts, 774 × 10^9^/L platelets, splenomegaly 3 cm below left costal margin and cytogenetic evidence of the Philadelphia chromosome. Bone marrow was consistent with CML in CP with marked eosinophilia (Fig. 1a). Additional chromosomal abnormalities were not detected. Multiplex-PCR [14] revealed the atypical *BCR::ABL1* transcript e19a2 (Fig. 1b). He was at intermediate risk according to the EUTOS long-term survival (ELTS) score. To specify the molecular follow-up of the atypical transcript e19a2, the *BCR::ABL1* fusion was confirmed by Sanger sequencing, and specific qPCR primers were applied to monitor response (Fig. 2a). *BCR::ABL1* transcript levels were calculated in relation to beta-glucuronidase (*GUSB*) transcripts and the ratios obtained over time were compared to the ratio at diagnosis in order to determine the individual molecular response (IMR) to asciminib [4] (Fig. 2b). Following first-line treatment with nilotinib within the ENEST1st trial (NCT01061177), an individual reduction of *BCR::ABL1* transcripts [4] by three log, approximating to an MMR, was achieved after 3 months. A four-log reduction was achieved after 9 months. However, loss of molecular and cytogenetic response occurred after 30 months; upon detection of a T315I mutation, second-line therapy with ponatinib was commenced. Prompted by newly diagnosed arterial hypertension, the dose of ponatinib was reduced from 45 to 30 mg daily. After 4 months of ponatinib, a three-log reduction was achieved again. Treatment was extended by adding pegylated interferon alfa-2b 35 µg weekly after 9 months with the aim of improving disease control, but without success.

Due to the patient’s young age and cardiovascular side effects of ponatinib [15], donor search was initiated and an allogeneic stem cell transplantation was discussed. At this time, the multicenter phase 1 trial of oral asciminib (ABL001) for patients with CML or Ph+ acute lymphoblastic leukemia (NCT02081378) was recruiting. After informed consent balancing the risks of transplantation vs. potential transformation of CML, treatment was switched to third-line asciminib 150 mg twice daily according to study randomization. Asciminib was well tolerated and after 4 weeks of treatment no measurable residual disease was detectable with five-log individual PCR sensitivity. After 5 months of therapy, grade 1 increase in serum lipase and amylase without clinical or radiologic correlates of pancreatitis occurred for the first time, so that after a 2-week break and normalization of enzyme activities, the dose of asciminib was reduced to 80 mg twice daily. Molecularly undetectable disease with five-log sensitivity continued, cardiovascular symptoms associated with ponatinib had disappeared. During the following treatment period of 7.3 years with asciminib, a sustained treatment response with almost consistently undetectable residual disease of CML was revealed in three-month PCR controls. Although there are no recommendations for CML with atypical transcripts, a discontinuation attempt was made after a shared decision-making with the patient. Within 18 months after treatment discontinuation, RT-qPCR monitoring did not reveal any evidence of *BCR::ABL1* expression with continuous five-log PCR sensitivity (*GUSB* transcript range 275,037–4,038,847) (Fig. 2b).

In general, CML patients with T315I mutation have an unfavorable prognosis. However, long-term outcome depends on phase of disease when T315I mutation occurs, with 5-year overall survival estimated at 70% in CP and 10% in blast phase [9, 11]. Even though ponatinib is highly effective in patients with T315I mutation, cardiovascular or thromboembolic side effects often necessitate dose modifications or discontinuation of therapy. The frequency of arterial hypertension as ponatinib-associated side effect, leading to change of treatment in the reported case, is reported to be 37% [15]. Due to its specific mechanism of action, asciminib shows fewer off-target effects and remains effective at higher doses even with T315I mutation [13]. However, available data on asciminib in patients with CML and T315I mutation are still limited. In the phase I clinical trial investigating asciminib monotherapy after prior treatment with at least one TKI, almost half of the patients with CML-CP and T315I mutation achieved MMR. Among ponatinib-naïve patients, about 68% achieved MMR against 35% of those pretreated with ponatinib. In patients switching from ponatinib to asciminib due to intolerance, MMR rates were notably higher than in those who were resistant, with around 57% and 15%, respectively. However, this analysis excluded three patients due to an atypical *BCR::ABL1* transcript, including the case reported here [13]. When switching to asciminib, the ratio *BCR::ABL1/GUSB* of this patient was 0.010% and T315I mutation was not detectable by Sanger sequencing. The described asymptomatic increase in lipase and amylase has been reported in 29% and 12% of patients treated with asciminib at the higher dose and in 5% and 6% of patients treated with the regular dose (80 mg daily), respectively [12, 13]. Following the study protocol, the dose was reduced in the reported case, but without any negative impact on treatment response. According to the European LeukemiaNet 2020 recommendations, treatment discontinuation in patients with atypical transcripts is not advised if quantitative PCR controls are routinely performed [6]. However, discontinuation can be carefully monitored in highly specialized laboratories so that samples from eligible patients should be allocated to them [4, 5]. Sequencing of the atypical *BCR::ABL1* transcript should be performed on a pre-treatment sample.

This is the first report of a CML patient with an atypical e19a2 *BCR::ABL1* transcript and T315I mutation in whom treatment with asciminib resulted in stable complete molecular remission with five log PCR sensitivity (MR^5^). In contrast to current recommendations [5], asciminib was discontinued after 7 years in individual MR^5^. Quantitative monitoring of residual disease was feasible using an individual approach and permitted the TFR attempt even after T315I triggered resistance. In order to provide access to innovative treatment options and to collect data on individual response patterns, patients with atypical *BCR::ABL1* transcripts who are potentially eligible for treatment cessation should be considered in prospective clinical trials.
